# Prediction of Postnatal Growth Failure among Very Low Birth Weight Infants

**DOI:** 10.1038/s41598-018-21647-9

**Published:** 2018-02-27

**Authors:** Soon Min Lee, Namhyo Kim, Ran Namgung, Minsoo Park, Kookin Park, Jihyun Jeon

**Affiliations:** 1 Department of Pediatrics, Yonsei University College of Medicine, Seoul, Korea; 2 Department of Pediatrics, Seonam University of Medicine, Myongji Hospital, Gyeonggi-do, Korea; 3 Department of Pediatrics, CHA Gangnam Medical Center, CHA University, Seoul, Korea

## Abstract

Postnatal growth failure (PGF) in preterm infants remains an important clinical issue. In this study, we analysed the incidence of PGF among very low birth weight (VLBW) infants and evaluated the risk factors for PGF based on the data of 2799 VLBW infants obtained from the Korean Neonatal Network database from 2013 to 2014. PGF was defined as a decrease in weight Z score between birth and discharge of more than −1.28 using the Fenton growth charts. Risk factors were evaluated in relation to birth weight for gestational age, namely small (SGA) or appropriate (AGA) for gestational age, using propensity score matching used for between-group differences. The overall incidence of PGF was 45.5%, with a rate of 68.9% in the SGA group and 36.2% in the AGA group. PGF was negatively correlated with gestation and birth weight; additionally, PGF was associated with a higher incidence of co-morbidities. Predictors of PGF in the SGA group were respiratory distress syndrome and days to attain 100 mL/kg of enteral feeding. The only predictor of PGF in the AGA group was days to attain 100 mL/kg of enteral feeding. Early initiation and aggressive progression of enteral nutrition may decrease the incidence of PGF.

## Introduction

The goal of nutrition for premature infants is to duplicate the in-utero growth rate and body composition of a foetus of the same gestational age^1^. However, achieving this weight gain goal is difficult and, consequently, preterm infants are frequently significantly underweight at the time of hospital discharge^2^.

Recently, the Vermont Oxford Network, which evaluates the postnatal growth of preterm infants, defined postnatal growth failure (PGF) as a discharge weight that is lower than the 10^th^ percentile for postmenstrual age. Among infants registered in this Network, the prevalence rate of PGF is 50.3%^3^. Kelleher reported that birth weight was significantly lower in low-birth-weight children with failure to thrive than in those without failure to thrive^4^. As well, small for gestational age (SGA) infants had poorer weight gain than appropriate for gestational age (AGA) infants^5^, and SGA infants remained at a lower body weight until the age of 3–6 years^6^.

Factors such as weight, gestational age, length of hospital stay, presence of respiratory distress syndrome (RDS), bronchopulmonary dysplasia (BPD), and sepsis have been associated with post-discharge growth and development in preterm infants^7^. According to a National Institute of Child Health and Human Development study on VLBW infants, nutritional intake deficits persist to some degree during the hospital stay, and infants with BPD, necrotizing enterocolitis (NEC), or late-onset sepsis demonstrate slower growth. Other studies have shown that infants with PGF were more likely to be SGA and to require significantly more days of mechanical ventilation, oxygen and time to regain birth weight that AGA infants^8,9^. Poor nutritional intake is common in VLBW in the early postnatal months^2^.

Growth impairment during early infancy can have permanent detrimental effects^2^. Impaired foetal and postnatal growth has been associated with neurodevelopmental delay, ischemic heart disease, impaired glucose tolerance, type-II diabetes mellitus, hypertension, and metabolic syndrome^10,11^. Measures of PGF for preterm infants have varied among studies, with no universally agreed upon criteria to define PGF. Generally, growth failure is considered when a baby’s weight is below the 10th percentile (≤−1.28 Z-score), but with different reference charts and variable postnatal periods having been used^12^. In this study, we defined postnatal weight growth as the change in weight Z score between birth and discharge, using the Fenton growth chart as a reference^13^.

Using this criterion, we evaluated the incidence of PGF among VLBW infants enrolled in the Korean Neonatal Network and investigated predictive factors of PGF through a comparison of PGF and non-PGF infants. In order to minimize errors due to the influence of SGA status, we also analysed the independent risk factors affecting postnatal growth in SGA and AGA groups separately, after propensity-score matching.

## Results

### Patients’ characteristics

**Figure 1:**
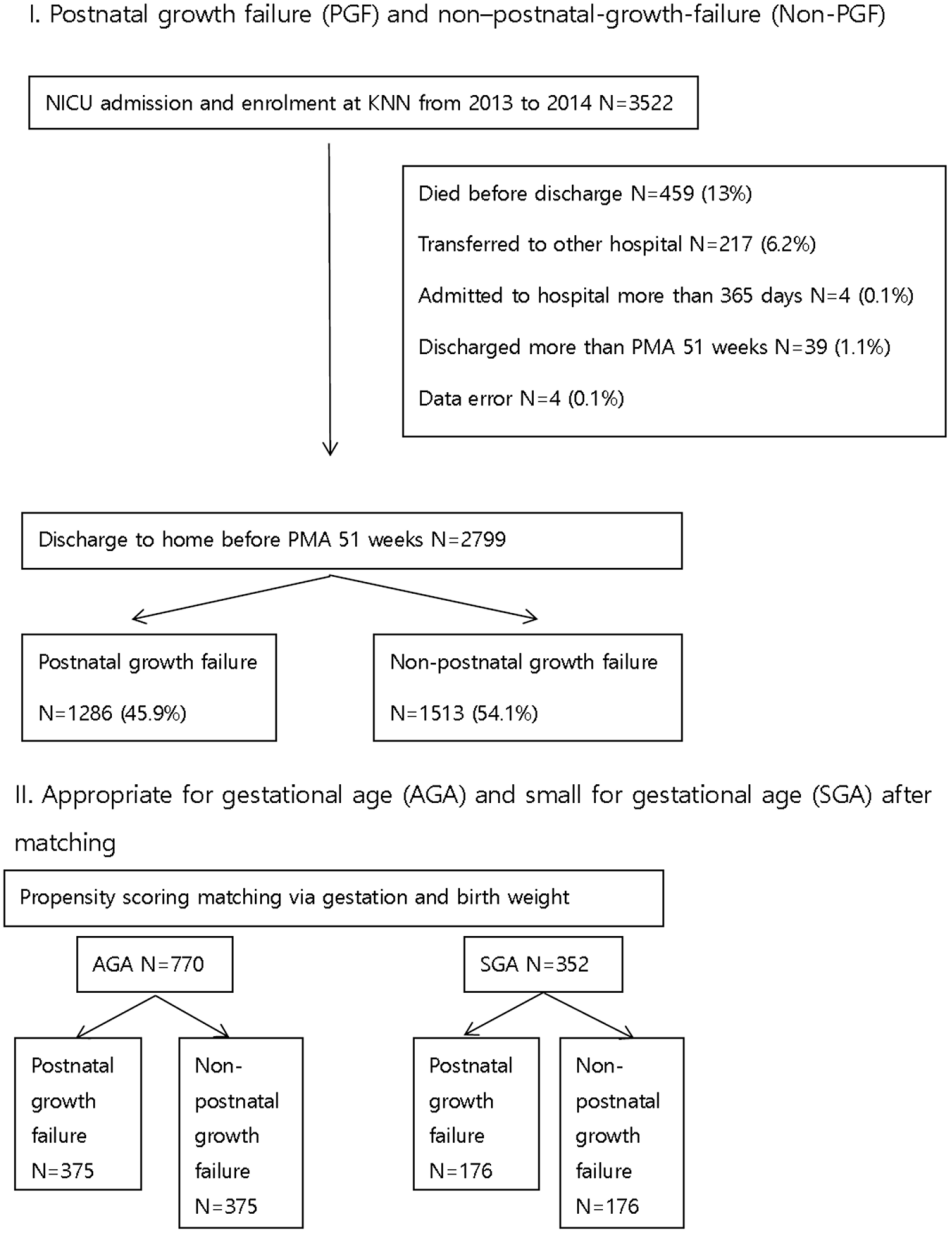
Flow Chart of study population.

Complete clinical data were available for 2799 patients, and the overall incidence of PGF was 45.9% (1286 of 2799; Fig. 1). The mean birth weight was 1132.91 g, with a mean gestational age of 28.98 weeks, respectively. The mean Z score of body weight at birth and at discharge was −0.45 and −1.78, respectively, with 29.7% of infants (830 of 2799) classified as SGA (Table 1).

**Table 1:** Differences in relevant clinical variables between the postnatal growth failure (PGF) and non-postnatal growth failure (Non-PGF) groups.

	PGF (N = 1286)	Non-PGF (N = 1513)	Total (N = 2799)	*p-*value
Oligohydramnios, n (%)	152 (10.0)	219 (17.0)	371 (13.3)	0.000
Multiple pregnancies, n (%)	450 (29.7)	517 (40.3)	967 (34.5)	0.141
IVF, n (%)	301 (19.9)	295 (22.9)	596 (20.3)	0.012
GDM, n (%)	135 (8.9)	105 (8.2)	240 (8.6)	0.499
PIH, n (%)	203 (13.4)	399 (31.0)	602 (21.5)	0.000
Male, n (%)	624 (41.2)	771 (60.0)	1395 (49.8)	0.211
C/sec, n (%)	967 (63.9)	1154 (89.7)	2121 (75.8)	0.507
SGA, n (%)	572 (44.5)	258 (17.1)	830 (29.7)	0.000
Gestation age, week	27.83 (2.46)	29.96 (2.70)	28.98 (2.81)	0.000
Birth weight, g	1053.61 (261.08)	1200.31 (233.70)	1132.91 (257.22)	0.000
Birth weight Z-score	−0.12 (1.02)	−0.73 (1.12)	−0.45 (1.11)	0.000
Weight at discharge, g	2440.47 (462.92)	2555.98 (598.63)	2502.91 (543.49)	0.000
Weight at discharge Z-score	−2.26 (1.20)	−1.37 (1.10)	−1.78 (1.23)	0.000
Apgar score 1-minute	4.51 (1.83)	5.30 (1.94)	4.94 (1.93)	0.000
Apgar score 5-minute	6.73 (1.63)	7.32 (1.55)	7.05 (1.61)	0.000
Hospital day, d	79.82 (28.54)	55.50 (25.94)	66.67 (29.74)	0.000

Data are presented as numbers of patients (%) or mean (SD).

PGF, postnatal growth failure; IVF, *in vitro* fertilization; GDM, gestational diabetes mellitus; PIH, pregnancy induced hypertension; C/Sec, caesarean section; SGA, small for gestational age.

### Clinical characteristics of the PGF and non-PGF groups

Significant differences between the PGF and non-PGF groups were identified for gestational age, birth weight and Apgar score. The incidence of PGF in SGA group was greater than in the AGA group (Table 1). As well, the incidence of co-morbidities (RDS; BPD; patent ductus arteriosus, PDA; NEC; intraventricular haemorrhage, IVH; periventricular leukomalacia, PVL; sepsis; and retinopathy of prematurity, ROP) was greater in the PGF than non-PGF group (Table 2).

**Table 2:** Comparisons of morbidities between the postnatal growth failure (PGF) and non-postnatal growth failure (Non-PGF) groups.

	PGF (N = 1286)	Non-PGF (N = 1513)	*p-*value
RDS, n (%)	1143 (88.9)	949 (62.7)	0.000
Air leak, n (%),	67 (5.2)	24 (1.6)	0.000
Pulmonary haemorrhage, n (%)	71 (5.5)	26 (1.7)	0.000
PDA treatment, n (%)	591 (46.0)	370 (24.5)	0.000
BPD (≥moderate), n (%)	453 (35.2)	294 (19.4)	0.000
IVH (≥Grade 2), n (%)	229 (17.8)	99 (6.5)	0.000
PVL, n (%)	127 (9.9)	85 (5.6)	0.000
NEC, n (%)	78 (6.1)	18 (1.2)	0.000
Sepsis, n (%),	326 (25.3)	172 (11.4)	0.000
ROP (≥stage 2), n (%)	187 (14.5)	82 (5.4)	0.000
Congenital anomaly, n (%)	32 (2.5)	15 (1.0)	0.003
Duration of invasive ventilator use, d	19.38 (23.03)	6.71 (12.76)	0.000
Duration of oxygen use, d	9.8 (13.96)	7.02 (11.65)	0.000
Duration of TPN, d	32.33 (23.75)	16.57 (13.84)	0.000
Days reaching above 100 cc/kg of enteral nutrition	29.44 (15.59)	25.39 (15.17)	0.000

Data are presented as numbers of patients (%) or mean (SD).

PGF, postnatal growth failure; RDS, respiratory distress syndrome; PDA, patent ductus arteriosus; BPD, bronchopulmonary dysplasia; IVH, intraventricular haemorrhage; PVL, periventricular leukomalacia; NEC, necrotizing enterocolitis; ROP, retinopathy of prematurity; TPN, total parenteral nutrition.

### Risk factors for postnatal growth failure among AGA infants

In the AGA group, after matching for gestational age, the incidence of oligohydramnios, RDS, drug therapy for PDA, and sepsis were higher in the PGF than non-PGF group. The duration of ventilation, hospitalization and parenteral nutrition, as well as the time needed to achieve 100 ml/kg of enteral feeding, were longer for the PGF than non-PGF group (Table 3). On Cox regression analysis, RDS (odds ratio [OR], 1.012, 95% confidence interval [CI] 1.008–1.050), and days to achieve 100 ml/kg of enteral feeding (OR 1.030, 95% CI 1.015–1.045) were retained as significant risk factors for PGF among AGA infants (Table 4).

**Table 3:** Comparisons of relevant patient demographics and morbidity between the postnatal growth failure (PGF) and non-postnatal growth failure (Non-PGF) groups specifically among infants with a weight appropriate for gestational age (AGA) after propensity score matching.

	PGF (N = 375)	Non-PGF (N = 375)	*p-*value
Oligohydramnios, n (%),	26 (6.9)	43 (11.5)	0.046
IVF, n (%)	99 (26.4)	76 (20.3)	0.058
PIH, n (%)	40 (10.7)	45 (12.0)	0.635
Chorioamnionitis, n (%)	93 (24.8)	99 (26.4)	0.654
Gestation age, week	28.32 (1.75)	28.32 (1.75)	1.000
Birth weight, g	971.28 (71.30)	971.80 (71.43)	0.997
Weight at discharge, g	2385.76 (430.09)	2834.82 (636.18)	0.000
Weight at discharge Z-score	−1.96 (0.87)	−0.61 (0.67)	0.000
Apgar score 1-minute	4.76 (1.90)	4.90 (1.93)	0.298
Apgar score 5-minute	6.94 (1.67)	6.93 (1.62)	0.923
Hospital day, d	69.68 (24.00)	65.80 (25.54)	0.015
RDS, n (%)	328 (87.5)	301 (80.3)	0.005
PDA treatment, n (%)	168 (44.8)	116 (30.9)	0.000
BPD (≥moderate), n (%)	106 (28.3)	100 (26.7)	0.661
IVH (≥Grade 2), n (%)	48 (12.8)	32 (8.5)	0.072
PVL, n (%)	37 (9.9)	29 (7.7)	0.350
NEC, n (%)	14 (3.7)	7 (1.9)	0.189
Sepsis, n (%),	80 (21.3)	47 (12.5)	0.001
ROP (≥stage 2), n (%)	121 (32.3)	102 (27.2)	0.137
Congenital anomaly, n (%)	9 (2.4)	2 (0.5)	0.065
Duration of invasive ventilator use	13.50 (19.01)	9.56 (14.28)	0.000
Duration of TPN, d	27.22 (20.46)	19.02 (14.79)	0.000
Days reaching above 100 cc/kg of enteral nutrition	24.24 (18.89)	17.43 (13.51)	0.000

Data are presented as numbers of patients (%) or mean (SD).

PGF, postnatal growth failure; IVF, *in vitro* fertilization; PIH, pregnancy induced hypertension; RDS, respiratory distress syndrome; PDA, patent ductus arteriosus; BPD, bronchopulmonary dysplasia; IVH, intraventricular haemorrhage; PVL, periventricular leukomalacia; NEC, necrotizing enterocolitis; ROP, retinopathy of prematurity; TPN, total parenteral nutrition.

**Table 4:** Risk factors for postnatal growth failure for the small for gestational age (SGA) and appropriate for gestational age (AGA) groups.

	*p-*value	Exp (B) (95.0% CI)
AGA		
RDS	0.043	1.012 (1.008–1.050)
PDA treatment	0.054	0.892 (0.476–1.006)
Sepsis	0.399	0.797 (0.471–1.350)
Oligohydramnios	0.405	1.267 (0.726–2.209)
Days reaching above 100 cc/kg of enteral nutrition	0.000	1.030 (1.015–1.045)
Duration of invasive ventilator use	0.266	1.009 (0.993–1.025)
SGA		
Days reaching above 100 cc/kg of enteral nutrition	0.012	1.028 (1.006–1.050)
Chorioamnionitis	0.586	1.238 (0.574–2.672)
Apgar score 5-min	0.296	0.897 (0.732–1.100)
PVL	0.073	0.133 (0.015–1.208)
Congenital anomaly	0.680	0.731 (0.164–3.248)
BPD	0.431	0.756 (0.377–1.516)

AGA, appropriate for gestational age; RDS, respiratory distress syndrome, SGA, small for gestational age; PVL, periventricular leukomalacia; BPD, bronchopulmonary dysplasia.

### Risk factors for postnatal growth failure among SGA infants

Among SGA infants, after matching for gestational age, the incidence of chorioamnionitis, BPD (moderate or severe), PVL, and congenital anomalies was higher in the PGF than non-PFG group. The Apgar score at 5 min was significantly lower in the PGF than non-PGF group. The duration of ventilation, hospitalization, parenteral nutrition, and achievement of 100 ml/kg of enteral feeding were also significantly longer in the PGF than the non-PGF group (Table 5). On Cox regression analysis, days to achieve 100 ml/kg of enteral feeding (OR 1.028, 95% CI 1.006–1.050) was retained as the only significant risk factor for PGF among SGA infants (Table 4).

**Table 5:** Comparisons of relevant patient demographics and morbidity between the postnatal growth failure (PGF) and non-postnatal growth failure (Non-PGF) groups specifically among infants small for gestational age (SGA) after propensity score matching.

	PGF (N = 176)	Non-PGF (N = 176)	*p-*value
Oligohydramnios, n (%),	37 (21.0)	24 (13.6)	0.085
IVF, n (%)	40 (22.7)	27 (15.3)	0.105
PIH, n (%)	82 (46.6)	92 (52.3)	0.387
Chorioamnionitis, n (%)	136 (77.3)	29 (16.5)	0.000
Gestation age, week	30.57 (2.43)	30.57 (2.43)	1.000
Birth weight, g	907.79 (147.82)	904.27 (147.33)	0.826
Weight at discharge, g	2179.36 (338.82)	2295.66 (416.45)	0.004
Weight at discharge Z-score	−3.56 (0.86)	−2.21 (0.62)	0.000
Apgar score 1-minute	4.85 (1.83)	5.03 (1.93)	0.308
Apgar score 5-minute	6.91 (1.52)	7.31 (1.52)	0.008
Hospital day, d	70.35 (27.09)	54.66 (24.26)	0.000
RDS, n (%)	120 (68.2)	108 (61.4)	0.213
PDA treatment, n (%)	49 (27.8)	52 (29.5)	0.818
BPD (≥moderate), n (%)	50 (28.4)	28 (15.9)	0.004
IVH (≥Grade 2), n (%)	10 (5.7)	4 (2.3)	0.180
PVL, n (%)	12 (6.8)	3 (1.7)	0.035
NEC, n (%)	10 (5.7)	4 (2.3)	0.180
Sepsis, n (%),	38 (21.6)	25 (14.2)	0.066
ROP (≥stage 2), n (%)	48 (27.3)	33 (18.8)	0.058
Congenital anomaly, n (%)	13 (7.4)	3 (1.7)	0.021
Duration of TPN, d	11.73 (16.57)	5.85 (11.68)	0.000
Duration of invasive ventilator use, d	26.97 (21.84)	18.20 (13.09)	0.000
Days reaching above 100 cc/kg of enteral nutrition, d	29.89 (25.78)	19.11 (14.26)	0.000

Data are presented as numbers of patients (%) or mean (SD).

PGF, postnatal growth failure; IVF, *in vitro* fertilization; PIH, pregnancy induced hypertension; RDS, respiratory distress syndrome; PDA, patent ductus arteriosus; BPD, bronchopulmonary dysplasia; IVH, intraventricular haemorrhage; PVL, periventricular leukomalacia; NEC, necrotizing enterocolitis; ROP, retinopathy of prematurity; TPN, total parenteral nutrition.

## Discussion

As advances in neonatal care have significantly improved the survival rate of VLBW preterm infants, continuous growth monitoring from the time of birth is a good predictor of clinical status, outcomes of treatment and nutritional status. In particular, since growth has a direct effect on neurological development, monitoring of growth patterns is very important for NICU clinicians^8,14^. Therefore, our analysis of postnatal growth patterns, using the data obtained from 55 NICU centres in Korea, is very important to ascertain an accurate epidemiology of PGF in Korea.

Despite differences in the definition of PGF, reference standards used and the population studied, PGF is a universal problem in preterm infants. The reported incidence of PGF, however, has varied depending on the reference for growth used. Using the Fenton growth curves, as per our study, one study reported an incidence of PGF of 32.6% among surviving infants of <31 week gestation, where PGF was defined by a discharge weight below the 10th percentile^15^. According to the 2013 data from the Vermont Oxford Network, 50.3% of VLBW infants were discharged with a body weight below the 10th percentile. In accordance with previous studies, our study identified an incidence of PGF of 45.9% among surviving VLBW infants in Korea, based on a definition of PGF as a Z-score change from birth to discharge of >−1.28.

Various factors affect postnatal growth. In a prospective study of dietary intake and growth, PGF was significantly correlated with prematurity^2^. In our study, PGF was associated with a lower gestational age and a significantly lower birth weight, with these infants having a lower Apgar scores at birth than non-PGF infants. The PGF group also developed RDS, air leak, and pulmonary hypertension more frequently, requiring a longer duration of ventilation support than the non-PGF group. Compared to the non-PGF group, the PGF group had a higher incidence of sepsis and NEC during the admission period, longer duration of total parenteral nutrition (TPN), and time to full oral feeding. In addition, as the period of oxygen-use became longer, outcomes of BPD, IVH and PVL were significantly more frequent than among non-PGF infants. Our findings were consistent with those of previous studies, in which PGF in VLBW infants has been associated with late-onset sepsis, surgical NEC, ROP, IVH, BPD, and a longer post-natal hospital stay^16,17^.

Recent studies have demonstrated a direct relationship between growth achieved before 40 weeks gestation and neurodevelopmental outcome^14,15^. In addition, long-term follow-up studies have shown that, preterm gestational age, length of hospital stay, and the presence of RDS, BPD, and sepsis all affect growth and development^7^. Considering the burden of neurodevelopmental impairments to families and societies, reducing the risk for PGF during hospitalization is important.

SGA infants had higher risk of adverse neonatal growth than AGA infants. To accurately identify risk factors of PGF, we used propensity score matching for birth weight and gestation, with an equal number of PGF and non-PGF infants in both AGA and SGA groups. The frequency of RDS, PDA treatment and sepsis were significantly higher among PGF than non-PGF infants in AGA group. In contrast, in the SGA group, chorioamnionitis as a maternal factor, and BPD and PVL as infant factors were significantly more common among PGF than non-PGF infants. The difference in risk factors for PGF between the AGA and SGA groups may be explained by intrauterine infection caused by chorioamnionitis in SGA, which appears to increase the risk for BPD and PVL. After adjusting for potential confounding variables, a longer time to reach full enteral feeding was a significant risk factor for PGF in both AGA and SGA infants. This finding supports previous studies which have suggested that aggressive nutrition should be used to prevent PGF^18–20^. Wilson *et al*. reported that aggressive nutritional regimen (enteral feeding + parenteral), initiated from post-natal day 1, reduced the incidence of PGF, 59% compared to 82% among preterm infants without nutritional intervention^18,21^. As well, the aggressive nutrition regimen lowered the incidence of adverse clinical or metabolic sequelae. The ESPGHAN Committee recommended enteral nutrient supplementation for preterm VLBW infants, including the use of fortified human milk and formula designed for premature infants, providing a reasonable range of energy intake of 110–135 kcal/kg/day^22,23^. Our data also identified achieving early full enteral nutrition and reducing the number of days of TPN as being necessary to prevent PGF.

The growth velocity of preterm infants has been evaluated using various methods, with the most frequently reported method after 2005 being the Z scores measured from birth to discharge^12^, with Fenton’s growth chart being the most commonly used reference for the calculation of Z-scores and, therefore, for the identification of PGF^9,12^. Accordingly, we used the Z-score change in body weight from birth to discharge to define PGF.

The limitations of our study need to be acknowledged. Complete and informative data on nutritional practices, specifically the composition and volume of feeding, as well as the timing and rate of feeding over the study period, were not available. However, the changes in growth cannot be explained about the patient characteristics or major morbidities independently. As well, growth was monitored only at birth and at discharge. Variations about the definition of PGF and the reference standards may have contributed confounding factors for the comparisons among studies. Differences in clinical factors and site variations could not be controlled for. Therefore, nutritional practice may have influenced the measurement improvement in weight gain.

In summary, we have described the occurrence of PGF in a large national neonatal cohort in Korea, providing the first report of national growth-outcome data for VLBW infants in Korea. We have also identified the duration of TPN and days to achieve enteral feeding of 100 ml/kg as independent risk factors of PGF among VLBW infants, which could play a role in reducing PGF. As a suggestion for future research, including long-term growth and neurodevelopmental outcomes of PGF infants may allow for the discovery and assessment of additional clinical predictors.

In conclusion, VLBW infants are at high risk for PGF and, therefore, greater attention to optimising nutritional intake, either enterally or parenterally, may decrease the incidence of PGF.

## Materials and Methods

### Study population

We performed a cohort study using prospectively collected data from 55 Korean Neonatal Network centres. Records of a total of 3522 VLBW infants, born between January 2013 and December 2014, and registered in the Korean Neonatal Network were reviewed. We excluded 723 VLBW infants who died or were transferred or discharged later than postmenstrual age (PMA) 51 weeks. A small number (0.01%) of the data records were incorrectly recorded, resulting in an unexplained error. The data of the remaining 2799 infants were included in our analysis. For analysis, we classified infants into the PGF and non-PGF groups, and compared maternal demographics and neonatal characteristics between groups. After propensity-score matching for gestational age and birth weight, PGF (n = 375) and non-PGF (n = 375) groups were analysed in infants born AGA and infants born SGA, PGF (n = 176) and non-PGF (n = 176) groups.

### Data collection and analysis

Trained research assistants prospectively gathered maternal, delivery and neonatal data up to the time of discharge, according to the operational procedures^24^. Gestational age was calculated according to maternal last menstrual period, early pregnancy ultrasound examination findings or the new Ballard score. Weight, head circumference and height were measured at birth and at discharge. To control for variation in gestational age and postnatal age, body weight was converted into a Z-score using the Fenton growth chart^25,26^. To ascertain the degree of postnatal growth, the Z-score at birth was subtracted from the Z-score at discharge. Postnatal growth was defined as the change in the Z-score for weight between birth and discharge. PGF was defined as a decrease in weight Z score between birth and discharge of more than −1.28 using the Fenton growth charts^13,27^. SGA was defined as birth weight below the 10^th^ percentile according to the intrauterine growth charts of Lubencho^28^.

Antenatal corticosteroid exposure was defined as the mother received one or more doses of any corticosteroid. Pulmonary haemorrhage was defined as massive and significant pulmonary haemorrhage that destabilized vital signs. PDA was defined as the use of medication or surgical treatment for therapeutic and/or prophylactic purposes. IVH was graded according to the method of Papile *et al*.^29^. NEC was classified according to the system of Bell *et al*.^30^. BPD was defined by the NIH classification^31^.

### Statistical analysis

Unadjusted comparisons of maternal demographics and neonatal characteristics between the non-PGF and PGF groups were performed using a chi-squared or Fisher’s exact test for categorical data and Student’s *t*-test for continuous data. To adjust for confounding effects of gestational age and birth weight, propensity-score matching was performed. Propensity-score matching is the method for controlling covariate imbalance that produces the selection bias. After matching, univariate analyses for continuous variables were performed using paired *t*-tests. Categorical variables were examined using a Cox regression analysis. Multivariate survival analyses, adjusted for the factors found to be significant on univariate analysis, were also performed to identify independent risk factors of PGF. The relationship between Z-score of the weight, height and head circumference at discharge and at birth were evaluated using Pearson’s correlation coefficients. The correlation between changes in Z score of the weight, height and head circumference and Z score at birth were analyzed. All statistical analyses were performed using SPSS software version 21.0 (IBM Corp., Chicago, IL, USA) and SAS version 9.2 (SAS Institute, Cary, NC, USA). P-values < 0.05 were considered to be statistically significant.

### Ethics statement

The data registration was approved by the institutional review board of every hospital participating in the Korean Neonatal Network (KNN). All protocols and methods in this study were carried out in accordance with relevant guidelines and regulations. Informed consent was obtained prospectively from parents of infants registered in the KNN.

The dataset generated and analysed during the current study are not publicly available due to the research of Korea Centres for Disease Control and Prevention, but are available from the corresponding author on reasonable request.
